# Monkeypox: Prevention Strategies and Challenges: Updated Review

**DOI:** 10.1002/hsr2.70640

**Published:** 2025-04-08

**Authors:** Abate Wondesen Tsige, Siraye Genzeb Ayele

**Affiliations:** ^1^ School of Pharmacy, Clinical Pharmacy Unit, Asrat Weldeyes Health Science Campus Debre Berhan University Debre Berhan Ethiopia; ^2^ Department of Midwifery School of Nursing and Midwifery, College of Health Sciences, Addis Ababa University Addis Ababa Ethiopia

**Keywords:** antiviral agents, clinical management, epidemics, monkeypox virus, Mpox vaccine, Mpox virus

## Abstract

**Background and Aims:**

The mpox virus, sometimes known as MPXV, is the cause of the disease mpox. The Monkeypox virus is a different Poxviridae family member from the orthopoxvirus (OPXV) group. Clades I and II are the two varieties of the Monkeypox virus. The mpox can spread from person to person through direct contact with infectious skin or other lesions, such as those on the mouth or genitalia. The mpox virus is spread from animal to people by bites or scratches, as well as through tasks including skinning, trapping, cooking, playing with carcasses, and eating animals. People with compromised immune systems, small children, those with a history of eczema, and pregnant women may be more susceptible to serious mpox illness. This review aimed to identify the challenges of mpox, treatment alternatives, and prevention modalities.

**Methods:**

This review addressed mpox virus etiology, epidemiology, risk factors, clinical presentations, clinical evaluation techniques, currently available treatments, and preventative measures. An analysis of the narrative data was conducted instead of a pooled analysis. Complete data published in English was included in a comprehensive literature search conducted across relevant databases pertaining to the mpox.

**Results:**

The recommended laboratory test for mpox is polymerase chain reaction detection of viral DNA. It is crucial to differentiate measles, scabies, herpes, syphilis, chickenpox, measles, bacterial skin infections, and allergies related to medications from mpox. The ability to distinguish between chickenpox and mpox is made by lymphadenopathy. The JYNNEOS vaccine, ACAM2000 vaccine, and MVABN also known as Imvamune vaccinations have now been investigated and authorized for usage during mpox epidemics in different locations. Tecovirimat, brincidofovir, and cidofovir which had previously shown promise against OPXV were used as antivirals during the 2022 outbreak.

**Conclusion:**

This review provides a brief overview of current vaccinations and antiviral medications that have been assessed for their potential as treatments since the mpox threat came into existence will be provided. It is useful to increase awareness and recognize the common clinical manifestations of mpox, diagnose, and its prevention methods. To effectively reduce the global transmission of mpox, the WHO should prioritize strategies that enhance early detection of the illness, careful administration of antiviral treatments, and focused vaccination initiatives for high‐risk groups or wider immunization in areas where the disease is common. Furthermore, it is crucial to establish preventive measures, conduct educational outreach, and implement robust healthcare policies.

## 1 Introduction

The increase in mpox in the Democratic Republic of the Congo (DRC) and an increasing number of African nations has been deemed a public health emergency of international concern under the International Health Regulations (2005) by WHO Director‐General [1].

Furthermore, the continuing Mpox outbreak has been formally declared a Public Health Emergency of Continental Security by the Africa Centers for Disease Control and Prevention (Africa CDC). This is the first time the agency has made such a statement since its founding in 2017. This statement gives the organization the authority to direct and oversee responses to major health emergencies in accordance with Article 3, Paragraph F of the Africa CDC Statutes [2]. Through the declaration, resources will be mobilized across affected countries, providing crucial funding; risk communication and community engagement will be strengthened; laboratory testing and surveillance will be boosted; and human resource capacities will be enhanced to respond to mpox through a One Health approach.

Monkeypox virus infection is the cause of mpox, often known as Monkeypox. The smallpox virus and this one belong to the same family. There is no connection between chickenpox and mpox [3]. The orthopoxvirus (OPXV) genus includes 10 species. Of these, the variola virus, which causes smallpox, is the most lethal due to its high virulence and 30% case fatality rate [2]. MPXV is a different Poxviridae family member that belongs to the OPXV group. A poxvirus infection usually causes lesions, skin nodules, or a widespread rash in humans and many other animals. Smallpox, which has been eradicated, and the variola virus are two more OPXV species that are harmful to humans. One of the main agents in the 1980 smallpox eradication effort was the vaccination virus, another OPXV that has been used to vaccinate humans. The virus has a 12‐day incubation period before spreading from person to person. Around 96% of the genomes of MPXV and the variola virus are similar. MPXV is an encapsulated, oval‐shaped, double‐stranded DNA (dsDNA) virus with a size range of 200–250 nm [4, 5]. The reason MPXV got its name is its initial detection in monkeys [6].

## 2 Etiology

As a zoonotic illness, mpox can infect both humans and animals. It is endemic, or frequently encountered, in regions of West and Central Africa. Small rodents, monkeys, and other local mammals have been identified to carry the mpox virus. The monkeypox virus, sometimes known as MPXV, is the cause of the disease mpox [7]. Clades I and II are the two varieties of the monkeypox virus. Greater severity of disease and death is caused by Clade I, formerly known as the Congo Basin (central Africa). Although the fatality rate from epidemics has decreased in more recent times, in certain cases up to 10% of the sick have died. Accordingly, Central Africa is home to Clade I. The kind of clade that started the global pandemic in 2022 was Clade II, the ex‐clade of West Africans. Clade II mpox infections are milder. The survival rate is higher than 99.9%. West Africa is home to Clade II [3].

## 3 Epidemiology

The virus that causes monkeypox was found in Denmark in 1958 after two epidemics of a disease resembling pox struck colonies of study monkeys. Although formerly called “monkeypox,” the disease's origin is still a mystery. Researchers believe nonhuman primates (such as monkeys) and African rodents may carry the virus and spread it to humans. In the Democratic Republic of the Congo, the first human case of mpox was identified in 1970. The disease later spread to rural parts of central and western Africa [8]. Following 1970, West Africa (clade II) and Central and East Africa (clade I) experienced intermittent cases of mpox. The mpox virus returned to Nigeria in 2017 and is now spreading among its citizens as well as among tourists visiting other countries.

The global mpox epidemic began in 2022. Before that, mpox occurrences were uncommon outside and were typically associated with travel or the importation of animals from areas where the disease is endemic. An unexpected and rapid outbreak of mpox occurred in May 2022, spreading quickly to all six WHO regions, the Americas, and Europe. About 87,000 cases and 112 deaths were reported from 110 nations. Through sexual networks, the global outbreak has moved from person to person and mostly harmed gay, bisexual, and other men who have sex with men. In 2022, mpox epidemics caused by Clade I MPXV transpired in refugee camps situated in the Republic of Sudan [7]. In accordance with contemporary nomenclature conventions, the disease was renamed by the WHO in 2022. According to those recommendations, illness names should limit needless negative consequences on trade, travel, tourism, or animal welfare and avoid upsetting any ethnic, national, regional, professional, or cultural groups. Its causative virus still goes by its legendary moniker [3, 7].

A total of 934 new laboratory‐confirmed cases of mpox and four deaths were reported to WHO from 26 countries in June 2024 (the most recent complete monthly disease monitoring data available), demonstrating the ongoing transmission of mpox worldwide. The WHO's most impacted regions were the African Region (567 cases), the Region of the Americas (175 cases), the European Region (100 cases), the Western Pacific Region (81 cases), and the South‐East Asia Region (11 cases), in order of the number of laboratory‐confirmed cases. Furthermore included in this issue is an update on the geographic spread of mpox in the WHO African Region during July and August of 2024, which was not yet included in global surveillance data as of June 30, 2024. Kenya, Rwanda, Uganda, and Burundi are the first four nations in Eastern Africa to disclose mpox cases. All of the cases have been sequenced to date from these countries and are clade I. They are all connected to the spreading outbreak in East and Central Africa [9].

A minimum of 12 African nations, encompassing hitherto unaffected states such as Burundi, Kenya, Rwanda, and Uganda, have reported cases of mpox. These nations have confirmed 2863 cases and 517 deaths in 2024 thus far, mostly in the DRC. From 7146 cases in 2022 and 14,957 cases in 2023, the number of suspected cases throughout the continent has risen to over 17,000, a notable increase [2].

In all, 1162 nations/territories/areas (hereinafter referred to as “countries”) in all six WHO Regions reported to WHO a cumulative total of 99 176 laboratory‐confirmed cases of mpox between 1 January 2022 and 30 June 2024, including 208 deaths. The United States of America (*n* = 33, 191), Brazil (*n* = 11, 212), Spain (*n* = 8, 084), France (*n* = 4, 272), Colombia (*n* = 4, 249), Mexico (*n* = 4, 124), the United Kingdom (*n* = 3, 952), Peru (*n* = 3, 875), Germany (*n* = 3, 857), and the Democratic Republic of the Congo (2, 999) are the ten nations that reported the highest cumulative number of confirmed cases globally between January 1, 2022, and June 30, 2024. The Democratic Republic of the Congo is now included in the top 10 nations with the greatest total number of confirmed cases worldwide for the first time. Together, these ten nations are responsible for 81% of all cases that are recorded worldwide. Globally, the median age of confirmed cases with available data is 34 years (interquartile range: 29–41 years), and 96.4% of those cases (87,189 of 90,410 cases) are males aged 18–44 continue to be disproportionately impacted by this outbreak and makeup 79.4% of reported cases, therefore the age and sex distribution of patients has remained steady over time, notably outside the African Region [9].

### 3.1 Pathogenesis of Mpox

**Figure 1:**
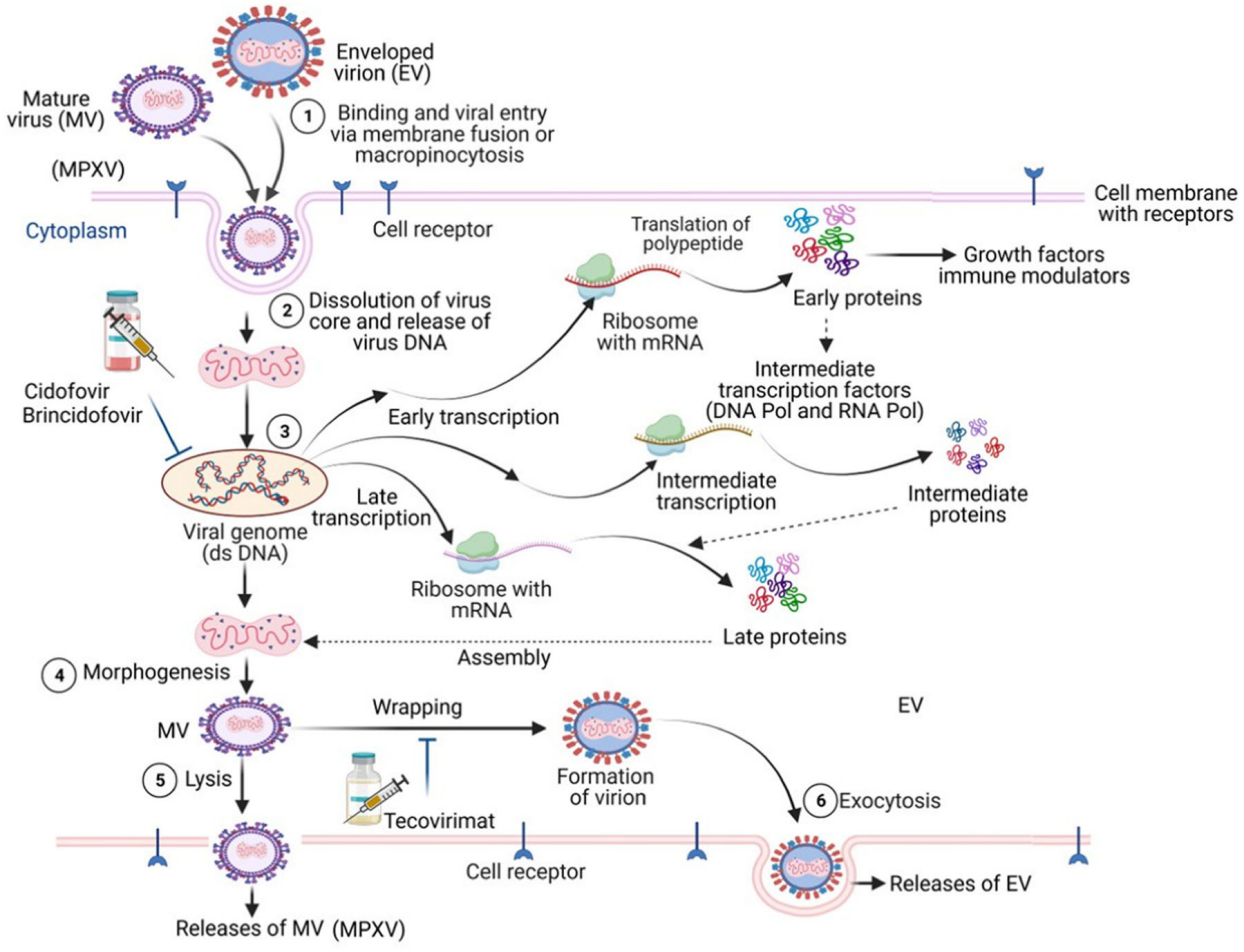
A schematic representation of the MPVX infection pathway in human cells. Membrane fusion or micropinocytosis are the methods by which MPVX mature viruses (MV) and enveloped viruses (EV) attach to and enter cells (step 1). As soon as MPXV enters the cytoplasm (step 2), it releases its genome and initiates step 3 transcription programs, which synthesize proteins and polypeptides that act as growth factors and immune modulators in the early, intermediate, and late stages. DNA pol and RNA pol are examples of intermediate transcription factors. Antiviral drugs, To prevent MPVX infection, cidofovir and brincidofovir specifically target this stage. Following the assembly of the MPVX genome and core components, morphogenesis (step 4) results in MV, which can either immediately release from the host cell membrane (step 5) or undergo wrapping to form EV, which is released by exocytosis (step 6). Tecovirimat's therapeutic target is the next step. MPXV executes its replication cycle in the host cell cytoplasm.

A dsDNA virus called MPXV is known to cause monkeypox in humans and several other animals [10] (Figure 1). The outer membrane, lateral bodies, lipoprotein‐based outer envelope, and core are the four components of the MPXV virion [11]. Its core contains the dsDNA genome and fibrils. With a core genomic region measuring 101 kb, the MPXV dsDNA genome has a size of 197 kb. Extra genomic elements include the coding region, brief tandem repeats (70 or 54 bp), exclusive ITR sections NR1 and NR2, and 6379 bp of terminal inverted repetition (ITR) at both terminal variable regions with an approximate 80 bp long hairpin loop [11, 12].

There are roughly 190 nonoverlapping open reading frames (ORFs) in the MPXV genome, of which four are found in the ITR sequence. Genes involved in viral transcription, replication, virion assembly, and release are found in the central genomic area of OPXVs, as has been routinely shown (Figure 1). The virulence genes on both ends of the MPXV genome are involved in immune evasion, primarily via interfering with signaling, antigen presentation and recognition, and cell death [13]. Less frequent genomic mutations in the MPXV are likely caused by the dsDNA structure and DNA polymerase 3′–5′ exonuclease activity. But according to reports, the genomes of MPXV variants from previous reports differ by about 50 single‐nucleotide polymorphisms (SNPs) in the 2022 variation [14].

Numerous investigations have demonstrated that RNA editing may hasten the changes and cause mutations in the MPXV genome [15]. Among the mechanisms thought to be involved are the enzymes known as apolipoprotein B mRNA‐editing catalytic polypeptide‐like 3 (APOBEC3) [16]. Compared to the MPXV‐2018 strain, the MPXV‐2022 strain features 46 novel consensus mutations, 24 of which are non‐synonymous variants, according to a phylogenetic study by Wang and colleagues [17]. Certain alterations that could aid the virus in eluding human immunity may become clearer with more research.

Enclosed viruses (EV) and mature viruses (MV) of MPVX attach and enter through micropinocytosis or membrane fusion. Cell surface binding was thought to be a role for viral proteins such as D8L, A27L, A34R, A26L224, and H3L, though this was not entirely evident. Later, MPXV releases its core into the cytoplasm of the host, where it contains essential components and enzymes that trigger the transcription of viral genes [18]. The host cell ribosomes mediate the translation of the early (polypeptides/proteins that act as growth factors and immunological modulators), intermediate (intermediate transcription factors including DNA pol and RNA pol), and late (proteins involved in MPVX genome assembly) proteins. Viral transcription is started by DNA‐dependent RNA polymerase [19]. Viral particles assemble to form intracellular MV, which matures into extracellular enveloped viruses (EV) during the stage of cell lysis and is maintained in the cytoplasm (Figure 1) [20]. It is also possible for the VP37 protein to mediate the MV's wrapping into an EV by covering it with a Golgi‐derived coating. Exocytosis then releases the EV [21]. Tecovirimat, an antiviral drug, targets the later step therapeutically.

### 3.2 Transmissions of Mpox

The mpox can spread from person to person through direct contact with infectious skin or other lesions, such as those on the mouth or genitalia. This includes breathing or talking face‐to‐face, touching or vaginally or anally, kissing or mouth‐to‐skin contact (oral sex or kissing the skin), respiratory droplets, or short‐range aerosols from prolonged close contact. Following that, the virus enters the body through the respiratory system, broken skin, or mucosal surfaces (such as the mouth, throat, eyes, genitalia, or anorectal). Mpox can infect sexual partners as well as other household members. Individuals who engage in several sexual relationships are more vulnerable [7].

The mpox virus is spread from animal to people by bites or scratches, as well as through tasks including skinning, trapping, cooking, playing with carcasses, and eating animals. Additional research is being conducted since the degree of viral circulation in animal populations is not fully understood. Mumps can be spread by contaminated items like clothes or linens, by sharp object injuries received in medical facilities, or in public places like tattoo parlors [2, 7].

Mpox can occasionally travel from animals to humans or from person to person. After smallpox was eradicated in 1980 and smallpox vaccinations were stopped globally, mpox gradually spread throughout central, east, and west Africa. There was a worldwide pandemic in 2022–2023. Although the virus's natural reservoir is unknown, a variety of tiny mammals, including monkeys and squirrels, are susceptible [7]. Transplacental and perinatal transmission are two further routes of transfer that have been documented [13]. It is advised to use condoms for up to 8 weeks after a diagnosis of Mpox to prevent transmission, as viral DNA has been found in some infected people weeks after contraction [22].

Direct contact with infected animals, close contact (including personal contact) with a mpox patient, and direct contact with contaminated items are the three main ways that the Clade I and II virus can spread [2, 3]. Mpox can happen to anyone. Contact with infected people, whether by touch, kissing, or sexual intercourse; items, such as contaminated sheets, clothes, or needles; and pregnancy, which can result in the virus being passed to the fetus. Until all wounds have healed and a new layer of skin has grown, people with mpox are contagious and can infect others [7].

## 4 Predisposing Factors

Not everyone who contracts mpox is at risk of serious illness; however, certain individuals such as those with compromised immune systems, small children, those with a history of eczema, and pregnant women may be more susceptible to serious illness [3]. Moreover, mpox problems can arise in those with compromised immune systems. The risk of serious illness and death from mpox is increased in individuals who have immune suppression from medication or medical disorders. Serious illness is more common in HIV‐positive individuals who are poorly managed or treated [7].

## 5 Clinical Presentations

Mpox symptoms can appear 1–21 days after exposure, however they typically do so within a week. When an individual's immune system is compromised, symptoms can linger longer than the usual duration of 2–4 weeks. A sore throat, fever, rash, headache, back discomfort, muscle aches, and low energy are typical signs of mpox. One typical mpox symptom is lymphadenopathy, or enlarged lymph nodes. There are certain individuals who can be affected and show no symptoms [7]. After your latest exposure, wait 21 days to check for mpox symptoms. See a doctor if you have any symptoms, such as a rash [23].

A rash may be the initial mpox symptom for some people, whereas other symptoms may appear initially in other people. First appearing as a flat sore, the rash progresses into a liquid‐filled blister that may hurt or itch. Lesions peel off, dry up, and crust over as the rash heals. A person may have one or a few skin lesions, whereas others may have hundreds or even thousands. These can develop anywhere on the body, including the face, mouth, throat, groin and vaginal regions, anus, and the palms and soles of the feet. Additionally, some sufferers experience excruciating rectum edema or pain and difficulties urinating [7]. The lesions at each anatomical site are usually the same size and maturation stage, unlike chickenpox [24].

## 6 Complications of Mpox

The main complications of Mpox include sepsis, inflammation of the brain (encephalitis), heart (myocarditis), rectum (proctitis), genital organs (balanitis) or urinary passages (urethritis), pneumonia, corneal infection with blindness, pain or difficulty swallowing, vomiting, and diarrhea resulting in severe dehydration or malnourishment, and death [7].

Ocular infections, neurological issues, pericarditis, complications related to mucosal lesions (oral, rectal, genital, and urethral), and complications from unchecked viral spread because of moderate to severe immune compromise particularly advanced HIV infection are among the severe manifestations of mpox [25].

## 7 Diagnosis of Mpox

### 7.1 Laboratory Assessments of Mpox

Testing ought to be made available to everybody who fits the description of a possible case. Clinical and epidemiological considerations should be considered when deciding whether to test, and this decision should be connected to an evaluation of the probability of infection [6, 26]. Hence, it's critical to take into account additional possible reasons for isolated skin lesions or a widespread rash. Some etiologies for similar‐looking skin lesions at different stages of development include bacterial skin infections, medication allergies, parapoxviruses (causing and related conditions), chancroid, herpes simplex virus, varicella‐zoster virus, chickenpox, molluscum contagiosum virus, enterovirus, measles, scabies, and treponema pallidum (syphilis) [27].

Nucleic acid amplification testing, such as real‐time or conventional polymerase chain reaction (PCR), is used in laboratories to confirm specimens from a suspected case. Generic nucleic acid amplification testing for the OPXV or specific testing for the monkeypoxvirus (MPXV) is preferred [6].

The recommended laboratory test for mpox is PCR detection of viral DNA. The finest diagnostic samples are obtained by forceful swabbing of the skin, fluid, or crusts straight from the rash. Oropharyngeal, anal, or rectal swabs can be used for testing in the absence of skin lesions. It is not advised to test blood. Since antibody detection techniques cannot distinguish between distinct orthopoxviruses, they may not be helpful [7].

The confirmation of MPXV infection is indicated by positive detection using an MPXV PCR assay in suspected cases, or by positive detection using an OPXV PCR assay followed by MPXV confirmation using PCR and/or sequencing. While MPXV‐specific confirmatory testing is ideal, OPXV PCR assay‐positive detection is thought to be enough for laboratory confirmation of suspected cases. Serological testing may be helpful to further explore prior infection for epidemiological purposes when the clinical presentation and epidemiology suggest an MPXV infection despite negative PCR results. False‐negative test results can occur for a variety of reasons, including subpar specimen quality, improper handling or shipment, or test‐specific technological issues such as unsuccessful DNA extraction. Genetic sequence data (GSD) can be used in conjunction with sequencing to aid in the diagnosis as well as gaining insight into the epidemiology, origins, and traits of the virus. For instance, GSD can reveal whether a virus is imported once or repeatedly from different sources [6].

The national reporting rules should be adhered to by laboratories. Immediately notify the national authorities of any test results, whether they are positive or negative. Part of the diagnosis and surveillance of this new virus is having prompt and accurate laboratory testing access for samples from patients who are under investigation. Each nation should have access to trustworthy testing, either domestically or by referral to foreign laboratories that are prepared and able to diagnose MPXV or OPXV [28].

Generally, for MPXV identification and type, diagnostic techniques based on nucleic acid amplification constitute the gold standard. By switching detection from laboratory testing to POCT, emerging technologies like CRISPR, isothermal amplification in conjunction with biosensors, and Gene‐Xpert greatly improve detection efficiency and stop the spread of disease brought on by patient clustering [29].

### 7.2 Clinical Assessments of Mpox

**Figure 2:**
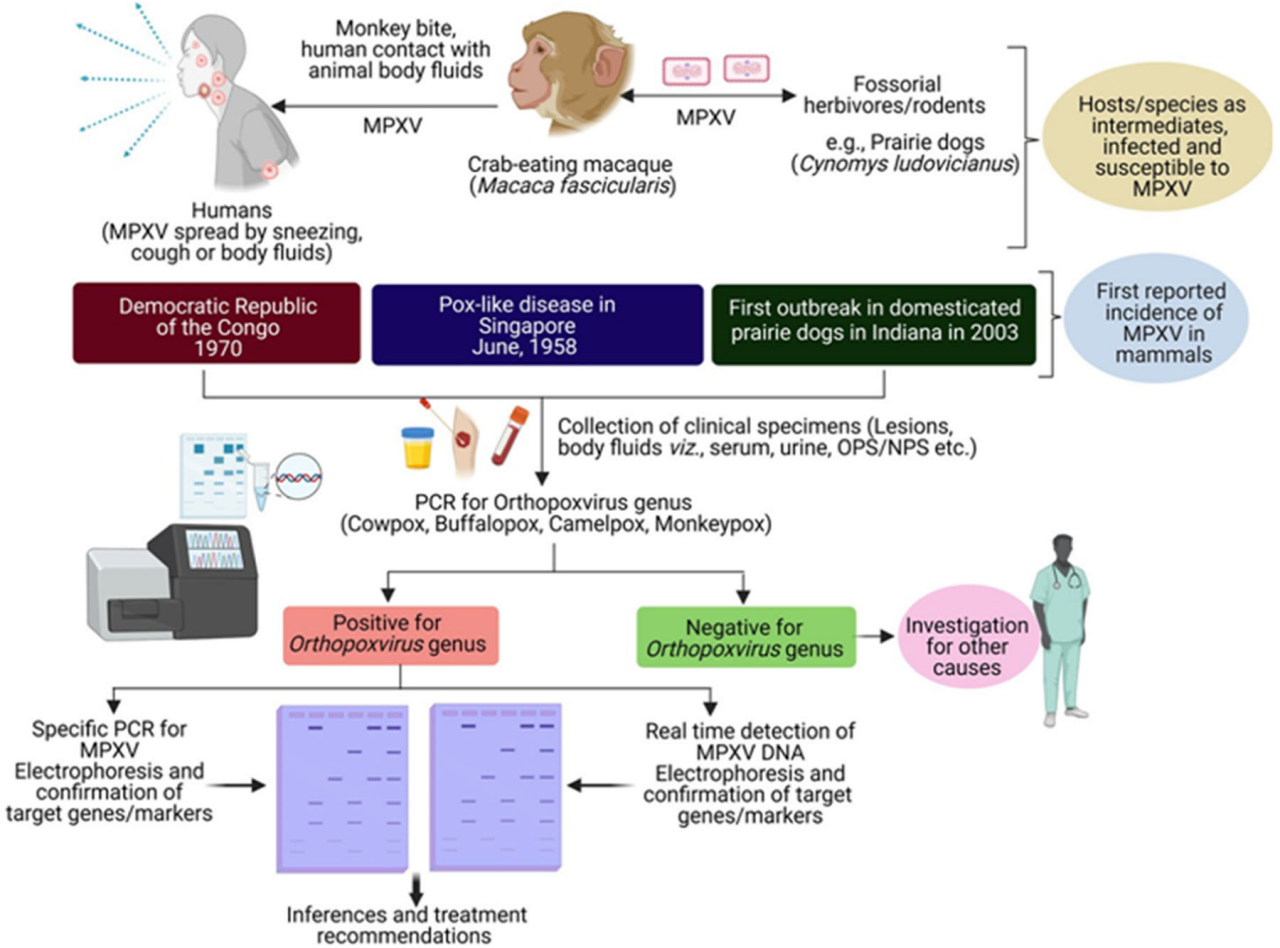
Diagrammatic representation of the clinical approaches used for MPXV diagnosis and evaluation.

An individual who has been diagnosed with MPXV should have a preliminary examination that focuses on the patient's past medical history. This should include details regarding the patient's travel to an area where the disease is common, their contact with other infected individuals, and the development of symptoms. Furthermore, it is important to record the patient's sexual history and if they have ever had the smallpox immunization. The eyes and oral mucosa should also be closely examined by medical personnel to assess the degree of the lesions. To accurately determine the possibility of an MPXV infection or exposure, a comprehensive physical examination must examine the neurological, gastrointestinal, respiratory, and cardiovascular systems [4] (Figure 2).

The combination of clinical signs, laboratory findings that support the diagnosis, and anamnestic data is used to diagnose monkeypox [30, 31]. As with other infections, mpox can be challenging to diagnose. It is crucial to differentiate measles, scabies, herpes, syphilis, chickenpox, measles, bacterial skin infections, and allergies related to medications from mpox. Another sexually transmitted virus, such as herpes, may coexist with mpox. Or a child who appears to have chickenpox may also have probable mpox [7]. The ability to distinguish between chickenpox and mpox during the prodromal phase is made possible by lymphadenopathy [32] (Figure 2).

## 8 Preventions and Treatment Mechanisms of Mpox

### 8.1 Preventions of mpox

Three vaccinations have now been investigated and authorized for usage during mpox epidemics in different locations [4].

### 8.2 JYNNEOS Vaccine

The JYNNEOS vaccine has previously received USFDA approval to treat MPXV infections. It has been sold under the trade name IMVANEX since the European Medicines Agency approved it in 2013 for use in the smallpox vaccination. In the US, the smallpox and MPXV vaccines are known as JYNNEOS, but in Canada, they are known as IMVAMUNE. The FDA granted IMVAMUNE a license in 2019; by 2022, it was licensed in Canada [33]. The JYNNEOS vaccine is non‐replicating because the live vaccinia virus employed in it reproduces poorly in human cells. It has demonstrated encouraging outcomes in animal models and clinical trials when used as a smallpox vaccine [34]. In contrast to the ACAM2000 immunization, JYNNEOS is safe for people with HIV and exfoliative skin conditions. The second subcutaneous injection of this vaccine, spaced 28 days apart, produces the highest level of immunity 14 days later. A detailed JYNNEOS usage protocol for children is being developed by the Centers for Disease Control and Prevention. Promising protective benefits of JYNNEOS against MPXV were demonstrated in several preclinical investigations [35, 36]. In macaques challenged with MPXV, it was also discovered to provide long‐lasting immunity and good protection [36]. In participants from the Democratic Republic of the Congo, Petersen and colleagues evaluated the safety, immunogenicity, and efficacy of the JYNNEOS vaccine. Two vaccination doses are administered to study participants, who are prospective cohort healthcare workers who are 18 years of age or older on days 0 and 28 [37].

### 8.3 ACAM2000 Vaccine

ACAM2000 has been authorized by the USFDA to treat MPXV, despite the fact that it is still recognized as a smallpox virus treatment through an enhanced Priority Investigational New Drug application. The ACAM2000 vaccination involves the replication of the vaccinia virus. This means that in patients with impaired immune systems, ACAM2000 must be used extremely carefully. Furthermore, given that ACAM2000 is associated with both pericarditis and myocarditis, questions have been raised regarding its tolerability [38]. ACAM2000 useful in avoiding smallpox viral infection in certain investigations; nevertheless, there is a dearth of information demonstrating its efficacy in MPXV infections.

### 8.4 MVABN Also Known as Imvamune

Two subcutaneous doses of the nonreplicating vaccination MVA‐BN must be given at least 4 weeks apart. The WHO states that mass immunization against mpox is not currently advised. Rather, primary preventative immunization is recommended for high‐risk groups, such as healthcare workers and people with multiple sexual partners. Immuno‐compromised people, expectant mothers, and children are among the other groups at risk of acquiring severe forms; however, immunization should only be given in situations when there is a demonstrable risk of exposure. All three vaccinations are good choices if there are no immune‐compromised conditions. On the other hand, the MVA‐BN vaccination needs to be reserved for people with serious immune system deficiencies, such as cancer patients or transplant recipients. For both primary and postexposure preventative vaccinations, this advice also pertains to women who are expecting or nursing [39].

Avoiding close skin‐to‐skin contact with individuals who have mpox‐like rash and animals that contain the virus are two methods to protect yourself and others against mpox. Another option is to get vaccinated [3].

If someone has mpox, cover lesions, wear a well‐fitting mask, refrain from touching other people, and follow at‐home treatment plan until a new layer of skin forms and all scabs fall off. Furthermore, be isolated for the length of the infectious period—that is, from the onset of symptoms until the lesions have healed and the scabs have fallen off—at home or in a hospital if necessary. It could be possible to stop the spread of lesions by covering them and donning a surgical mask around other people. Although condom use during intercourse can lessen the chance of contracting mpox, it cannot stop the virus from spreading orally or from the skin to skin to‐skin interaction [7].

When a person comes into touch with someone who has the mpox, they should receive the vaccination within 4 days (or up to 14 days if there are no symptoms). Getting vaccinated against mpox is advised for those who are at high risk, particularly in the event of an outbreak. Men who have intercourse with men, those who have several sexual partners, health workers who may be exposed, and sex workers are among those who fall into this category [7]. It appears that the smallpox vaccination provides around 85% protection against mpox, and those who have already received the vaccination tend to be less prone to infection [40, 41].

The five steps listed below can help prevent contracting monkeypox: two doses of the JYNNEOS vaccine 4 weeks apart were given; avoid close skin‐to‐skin contact with animals and people who have a rash similar to that of mpox; avoid handling materials and objects that a person who has mpox has handled; and wash hands [23].

## 9 Treatments of Mpox

As of right now, no medication has been licensed expressly to treat illnesses caused by the mpox. Most mpox patients who do not have a skin condition and have healthy immune systems will recover with supportive care and pain management without the need for medical intervention [25].

It is a self‐resolving illness that usually goes away on its own without any medical intervention [38]. On rare occasions, supportive care is required, particularly when dehydration has developed coupled with changes in renal function and/or problems related to microorganisms [39]. Antiviral therapy, however, can be extremely beneficial and occasionally even life‐saving in a few specific circumstances. A more gradual course of the disease marked by numerous localized lesions that may lead to problems may be experienced by immune‐compromised people, particularly those with reduced immune systems like PLWH [40]. As such, drugs that had previously shown promise against orthopoxviruses were used as antivirals during the 2022 outbreak [41]. It is noteworthy that among the agents utilized were tecovirimat, brincidofovir, and cidofovir (Table 1).

**Table 1:** The three antivirals that are effective against Mpox have different pharmacological characteristics [41].

MPX EC50	Tecovirimat	Brincidofovir	Cidofovir
	0.07–0.16 µM	0.07–1.2 µM	27–78 µM
The working mechanism	lowers the amount of extracellular virus produced by inhibiting the viral protein p37, which is involved in the last stages of the virus's maturation.	An inhibitor of DNA polymerase	An inhibitor of DNA polymerase
T 1/2	18–26 h	19.3 h (CDV diphosphate 113 h)	3.2–4.4 h (intracellular t 1/2
Protein binding	77%–82%	> 99.9%	< 6%
Elimination	23% of feces (mostly as visible pharmaceuticals); 73% of urine (mostly as metabolites)	Metabolites are expelled as bile and urine	Ninety percent of the whole dosage is eliminated by the kidneys through the major renal pathway.
Preparation	200 mg in capsule form and an injection of 10 mg/mL	Tablet (10 mg) and oral suspension (10 mg/ml)	Injection
Administration	Po or IV	Oral only	IV
Formulation	Oral (taken within 30 min following a full meal): 200 mg Q12h for 13–24 kg, 400 mg Q12h for 25–39 kg, 600 mg Q12h for 40–119 kg, and 600 mg Q8h for 120 kg and above.	For less than 10 kg, the suspension is given once every week in two doses on Days 1 and 8; for 10 kg to less than 48 kg, the suspension is given once every week in two doses on Days 1 and 8; for 48 kg and more, the dosage is given once every week in two doses on Days 1 and 8 (20 mL or one tablet); for tablets, the dosage is 200 mg on Days 1 and 8 (no food necessary).	No conclusive poxvirus dose data; 5 mg/kg IV once weekly for two weeks (may repeat 5 mg/kg every other week after that). Appropriately scheduled intravenous rehydration with probenecid and regular saline: 1 g dosages two and 8 h after the cidofovir infusion, and 2 g PO 3 h before each dose.
	Injection: 6 mg/kg Q12h over 6 h for kg 3–34; 200 mg Q12h over 6 h for kg 35–119; Above 120 kg: 300 mg every 12 h for 6 h.		
Treatment period	14 days	2 doses (Day 1 and 8)	Limited data; the Mpox model produced a single dose of 5 mg/kg.
FDA approval	To treat human smallpox, adults and children weigh at least 3 kilograms	Using adults, children, and newborns to cure human smallpox	Treatment of CMV retinitis in patients with AIDS
Kidney dose optimization	No change in dosage for the capsule, and injectable contraindication when CrCl < 30 mL/min	No modification in dose is necessary	Not advised if CrCl is less than 55 mL/min.
Liver dose optimization	Not required	Not required	No data
Use during pregnancy	Safe in animals; no human data	possible harm to fetuses based on data from animals. There is no human data available.	Not advised in pregnancy
Adverse events(most common)	Vomiting, headaches, nausea, and stomachaches	vomiting, elevated bilirubin or transaminases (2%–7%), and diarrhea. may permanently reduce an animal's ability to reproduce	Probenecid: hypersensitivity responses, rash, nausea, and vomiting; neutropenia, reduced ocular pressure, nephrotoxicity, and dose‐dependent tubular damage (Fanconi‐like syndrome).

Abbreviations: BID, twice daily; CDV, cidofovir; CMV, cytomegalovirus; CrCl, creatinine clearance; EC50, or half‐maximum effective concentration; FDA, food and drug administration; t1/2, half‐life.

### 9.1 Tecovirimat (Also Known as TPOXX, St‐246)

Smallpox, cowpox, and mpox can all be prevented by the antiviral compound tecovirimat [42]. By suppressing the VP37 protein, which is essential for the production of envelopes, it prevents the spread of extracellular viruses [43]. Despite smallpox being declared eradicated in 1980, the US FDA approved tecovirimat in 2018 for its use in treating the illness [44, 45]. Preclinical investigations have confirmed the efficacy of tecovirimat in treating mpox [46, 47, 48]. Tecovirimat showed the ability to considerably reduce mortality rates in animals exposed to mpox in these animal‐based studies, with survival rates of at least 90%. However, if treatment commenced later than intended, its effectiveness in preventing death decreased [49].

Both adults and children can utilize it if they weigh at least 13 kg. In addition, an intravenous formulation is offered for individuals who experience difficulty swallowing. Formulations taken orally are advised [50].

To test the safety and effectiveness of tecovirimat for treating clade I mpox in adults and children, the PALM007 trial was initiated in October 2022 by the National Institute of Allergy and Infectious Diseases, a division of the US. National Institutes of Health, and the National Institute for Biomedical Research of the DRC. At two sites in the Democratic Republic of Congo, 597 participants with laboratory‐confirmed mpox were enrolled in the randomized, placebo‐controlled research. Participants in the study were admitted to a hospital for at least 14 days, during which time they were intensively observed for safety and the resolution of mpox lesions. They were randomly assigned to receive either tecovirimat or a placebo. Supportive care was given to each participant, which included hydration, food, and secondary infection therapy. According to preliminary results published in August 2024, tecovirimat did not lessen the length of mpox lesions in adults or children with clade I mpox in the DRC. Nonetheless, the study's 1.7% overall mortality was much lower than the 3.6% or higher reported mpox mortality among cases analyzed collectively over the whole DRC, indicating that hospitalization and excellent supportive care can enhance the prognosis of mpox patients [25].

In cases of severe disease (hemorrhagic disease, multiple confluent lesions, sepsis, encephalitis, ocular or periorbital infections, or other conditions requiring hospitalization), involvement of anatomic areas that may have serious consequences (such as scarring or strictures), and severe infections (such as secondary bacterial skin infections), especially those that necessitate surgical intervention (e.g., debridement), the use of trecovirimat should be taken into consideration.

Tecovirimat should also be considered for use in people who are at high risk for severe disease including humans currently experiencing intense immunocompromise due to conditions consisting of: HIV/AIDS, leukemia, lymphoma, generalized malignancy, solid organ transplantation, remedy with alkylating dealers, antimetabolites, radiation, tumor necrosis factor inhibitors, or excessive‐dose corticosteroids, being a recipient of a hematopoietic stem cellular transplant < 24 months publish‐transplant or ≥ 24 months but with graft‐versus‐host ailment or sickness relapse, or having autoimmune disease with immunodeficiency as a clinical factor; pediatric populations, mainly patients younger than 8 years of age; being pregnant or breastfeeding girls; human beings with the subsequent situations: atopic dermatitis, eczema, burns, impetigo, varicella zoster virus contamination, herpes simplex virus contamination, intense acne, intense diaper dermatitis with extensive regions of denuded skin, psoriasis, or darier ailment (keratosis follicularis).

If patients with mpox need more than supportive care, tecovirimat is usually the first treatment that should be taken into consideration. When treating mpox in specific patients who require a different or additional course of treatment from tecovirimat, two additional treatments that are accessible from the Strategic National Stockpile (SNS) are brincidofovir and Vaccinia Immune Globulin (VIGIV). While treating MPXV infections, extra therapeutics can be used at the side of tecovirimat or as an opportunity remedy in specific situations, inclusive of ocular infections, people with existence‐threatening or prolonged mpox signs due to, for example, extreme immune‐compromise (HIV CD4 mobile count < 200 cells/mm3 or other similar intense immune‐compromise), sufferers experiencing clinically enormous ailment progression even as on tecovirimat, or sufferers experiencing recrudescence (first improvement accompanied by way of worsening of the disease after a preliminary period of development whilst on tecovirimat), patients for whom there may be a challenge that the virus affecting them may be immune to tecovirimat, along with while new lesions seem notwithstanding more than weeks of tecovirimat remedy, and people allergic to or otherwise not able to get hold of tecovirimat. Depending on a range of clinical and other factors, each person must make an individual decision about whether and when to employ these additional or alternative therapies [25].

### 9.2 Brincidofovir (Also Known as CMX001 or Tembexa)

The FDA has approved the prodrug brincidofovir, a derivative of cidofovir, to treat smallpox in adults, children, and newborns. Research on brincidofovir's efficacy in treating MPXV infection in humans is lacking. But investigations conducted in vitro and on animals have demonstrated its efficacy against orthopoxviruses. You shouldn't use cidofovir and brincidofovir at the same time [25].

Compared with cidofovir, brincidofovir has the advantage of causing less side effects, such as nephrotoxicity, which has been observed in cases of intravenous treatment in humans and animals [51]. Taking it orally as a tablet or suspension has an additional benefit over cidofovir, especially for people who have trouble swallowing. Brincidofovir showed promise against a variety of DNA viruses, most notably adenoviruses and cytomegalovirus (CMV), with a particular emphasis on poxviruses like mpox [52].

Bicidofovir is the second medication to be formally approved by the FDA for treatment against smallpox in June 2021, following tecovirimat [53, 54]. Still, because it is not feasible to perform comprehensive and carefully regulated field trials, the efficacy of brincidofovir in treating smallpox in humans has not been determined. When a physician requests and receives an FDA‐approved single‐patient, emergency‐use Investigational New Drug (e‐IND), brincidofovir is made available through the Strategic National Stockpile (SNS) for the treatment of mpox. Adults and pediatric patients, including newborns, who satisfy the following requirements may be eligible to receive brincidofovir under an e‐IND for the treatment of human monkeypox [55]. Being identified with excessive contamination or being at excessive hazard of developing an excessive contamination, as well as assembly any of the following criteria: showing a clinically significant worsening of the illness even as receiving tecovirimat treatment, or experiencing a recurrence of the contamination (a preliminary duration of development accompanied by a deterioration) after a period of development whilst receiving tecovirimat; being otherwise ineligible for or having a medical situation that stops using oral or intravenous tecovirimat, being otherwise ineligible for or having a contraindication for oral or intravenous tecovirimat.

Since most patients receiving brincidofovir have significantly impaired immune systems, they also need to take tecovirimat in addition to brincidofovir. It can be a concerned because using brincidofovir is associated with an increase in liver enzymes. This is why careful monitoring of hepatic function is required while using it [56].

### 9.3 Vaccinia Immune Globulin Intravenous (VIGIV)

The FDA has authorized VIGIV for the management of side effects resulting from vaccination. It is not, however, authorized for the treatment of mpox. There is no information on how well VIGIV treats MPXV virus infections in humans. There is no evidence that using VIGIV to treat mpox is beneficial, and it is uncertain if using VIGIV will help someone who has a severe infection. Healthcare professionals might, however, take it into consideration in extreme situations where there may be a risk of the formation of a strong antibody response. When a person is exposed to MPXV and has a severe impairment in T‐cell function, vaccination against smallpox or mpox is not advised as a preventive measure. In this case, VIGIV may be used. Individuals who are given VIGIV usually also receive tecovirimat and either cidofovir or brincidofovir simultaneously. By case‐by‐case request, clinicians can obtain VIGIV from the CDC [25].

### 9.4 Cidofovir (Also Known as Vistide)

FDA‐approved antiviral drug cidofovir is available for purchase as an injectable to treat CMV retinitis in AIDS patients. Information about cidofovir's efficacy in treating MPXV infection in humans is not readily available. Compared to cidofovir, brincidofovir, a prodrug of cidofovir, may have a better safety profile. Compared to cidofovir treatment, brincidofovir treatment for cytomegalovirus infections has not been associated with serious renal damage or other side effects. Since nearly all cidofovir patients for mpox have significantly impaired immune systems, tecovirimat must be used in conjunction with cidofovir [25].

As an extensive‐spectrum antiviral, cidofovir is powerful against a wide range of viruses (fifty‐six). Adenoviruses (of the Adenoviridae family), poxviruses (of the Poxviridae own family), papovaviruses (of the Papovaviridae circle of relatives) which include Papillomavirus (HPV) and Polyomavirus, varicella‐zoster virus (VZV), CMV, and Epstein‐Barr virus (EBV) are the various viruses in this spectrum [57]. The nucleotide counterpart of cytidine monophosphate is called cidofovir. It works by specifically blocking the creation of viral DNA [58]. In addition to competing with the enzyme's natural substrate, it inhibits the viral DNA polymerase to accomplish this. Cidofovir is marketed as an injectable and was approved by the FDA in 1996 for the treatment of CMV retinitis in AIDS patients. There has been some evidence of efficacy against poxviruses from a few in vitro and animal investigations. Some doctors used off‐label cidofovir, especially for severe or difficult types of Mpox, due to the unavailability of both tecovirimat and brincidofovir in many Western nations during the 2022 Mpox outbreak, with some encouraging results [59]. In actuality, four guys with severe Mpox cases were described in a case series that came from the San Raffaele Scientific Institute in Milan, Italy [60]. From June through August of 2022, cidofovir was administered intravenously to each of these people once.

In every case, the writers of these reports saw a quick improvement in a few short days. This was demonstrated by a decrease in the number of lesions and crust formation in addition to the remission of the first symptoms. Therefore, more cidofovir administrations were not required. Interestingly, no side effects or new symptoms appeared when cidofovir was administered in these instances.

**Figure 3:**
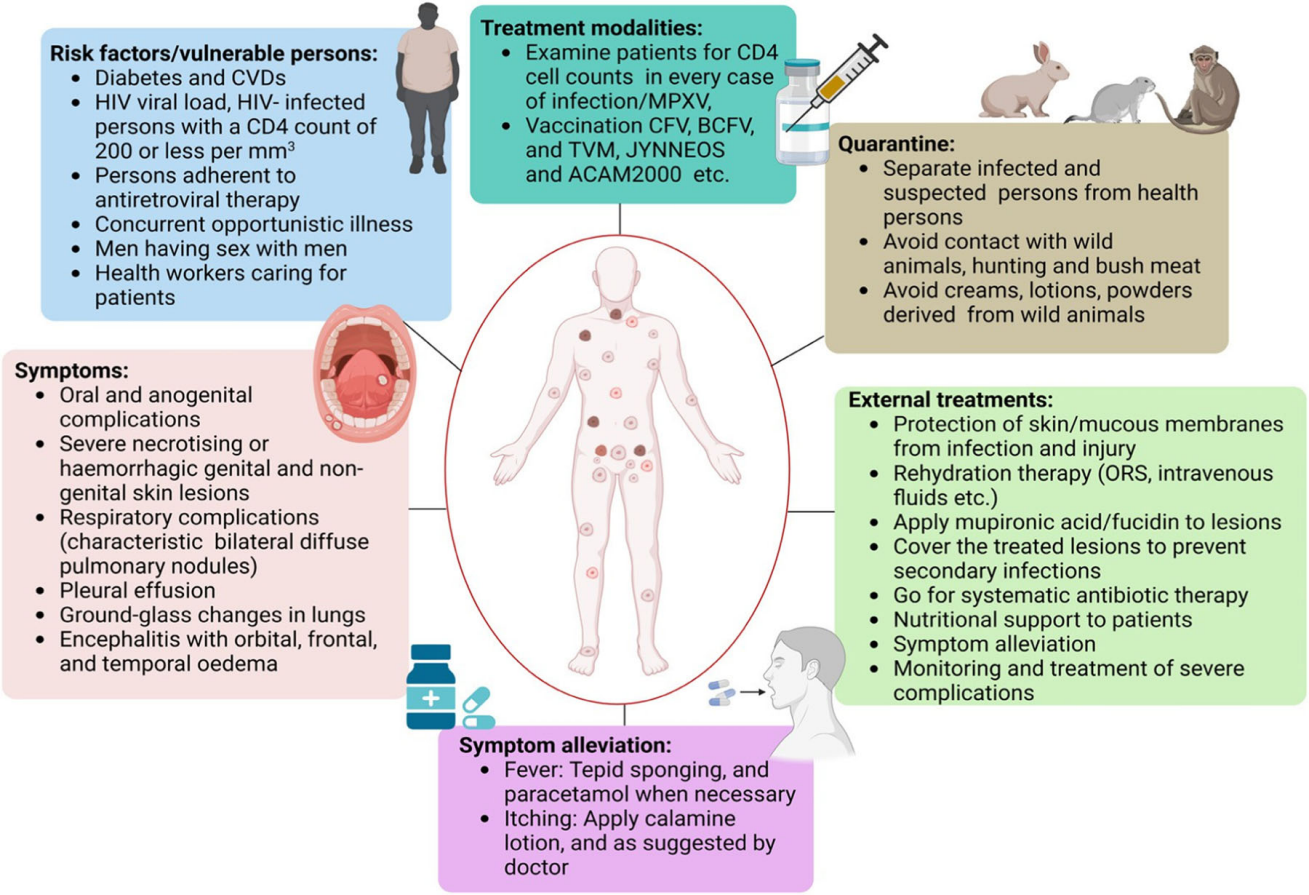
A diagram that illustrates the many MPXV therapy approaches, including taking into account the patient's comorbidities to determine risk factors and going over the patient's symptoms. It aids medical personnel in choosing the best course of action and treatment regimens, which may involve antiviral medications, vaccinations, isolation, outside therapy, and careful consideration of symptom relief.

Vaccincinia immune globulin is a hyperimmune globulin that the USFDA has licensed for the treatment of more severe vaccine‐related side effects [38]. Prioritizing nutrition and preventing skin problems should come first. For cutaneous lesions, topical petroleum jelly treatment and oral antihistamine administration work well. Buccal infections can be treated locally by using anesthetic gels, which often contain steroids, antimicrobials, and antihistamines. Under certain circumstances, treating painful vaginal lesions may benefit from the use of local anesthetics, nonsteroidal anti‐inflammatory drugs, and opioid analgesics [4] (Figure 3).

### 9.5 Challenges in the Treatment and Prevention of Mpox

As of now, there are no definitive vaccines or medications available for mpox. First‐line therapy of mpox is supportive care. The accessibility of vaccines and treatment is constrained by factors such as public awareness, the state of healthcare infrastructure, and the global response to disease outbreaks. This is the main actual challenge in the treatment and prevention of mpox.

## 10 Conclusions and Future Directions

This review provides a brief overview of current vaccinations and antiviral medications that have been assessed for their potential as treatments since the mpox threat came into existence will be provided. It is useful to increase awareness and recognize the common clinical manifestations of mpox, diagnose, and its prevention methods.

To effectively reduce the global transmission of mpox, the WHO should prioritize strategies that enhance early detection of the illness, careful administration of antiviral treatments, and focused vaccination initiatives for high‐risk groups or wider immunization in areas where the disease is common. Furthermore, it is crucial to establish preventive measures, conduct educational outreach, and implement robust healthcare policies.

In the future, the WHO and scientific communities will place a strong emphasis on research and public health initiatives to address mpox more efficiently and effectively.

## Author Contributions

**Abate Wondesen Tsige:** writing – original draft, funding acquisition, investigation, conceptualization, methodology, validation, visualization, writing – review and editing, software, formal analysis, project administration, data curation, supervision, resources. **Siraye Genzeb Ayele:** conceptualization, investigation, funding acquisition, writing – original draft, methodology, validation, visualization, writing – review and editing, software, formal analysis, project administration, data curation, supervision, resources.

## Ethics Statement

The authors have nothing to report.

## Consent

The authors have nothing to report.

## Conflicts of Interest

The authors declare no conflicts of interest.

## Transparency Statement

The lead author Siraye Genzeb Ayele affirms that this manuscript is an honest, accurate, and transparent account of the study being reported; that no important aspects of the study have been omitted; and that any discrepancies from the study as planned (and, if relevant, registered) have been explained.

## Data Availability

Data available on request from the authors.
